# A New Collaborative Recommendation Approach Based on Users Clustering Using Artificial Bee Colony Algorithm

**DOI:** 10.1155/2013/869658

**Published:** 2013-11-27

**Authors:** Chunhua Ju, Chonghuan Xu

**Affiliations:** ^1^Center for Studies of Modern Business, Zhejiang Gongshang University, Hangzhou 310018, China; ^2^College of Computer Science & Information Engineering, Zhejiang Gongshang University, Hangzhou 310018, China; ^3^College of Business Administration, Zhejiang Gongshang University, Hangzhou 310018, China

## Abstract

Although there are many good collaborative recommendation methods, it is still a challenge to increase the accuracy and diversity of these methods to fulfill users' preferences. In this paper, we propose a novel collaborative filtering recommendation approach based on *K*-means clustering algorithm. In the process of clustering, we use artificial bee colony (ABC) algorithm to overcome the local optimal problem caused by *K*-means. After that we adopt the modified cosine similarity to compute the similarity between users in the same clusters. Finally, we generate recommendation results for the corresponding target users. Detailed numerical analysis on a benchmark dataset *MovieLens* and a real-world dataset indicates that our new collaborative filtering approach based on users clustering algorithm outperforms many other recommendation methods.

## 1. Introduction

Nowadays, the Internet continues growing at an exponential rate in its size and complexity. For the users of numerous web sites it becomes increasingly difficult and time consuming to find the information they are actually looking for. As a consequence, how to efficiently help users filter out the unwanted information and find what is really useful for them is a challenging problem for information filtering. Recommender systems have proven to be an effective method to deal with the problem of information overload in finding interesting products. They not only help decide which products to offer to an individual customer but also increase cross-sell by suggesting additional products to the customers and improve consumer loyalty because consumers tend to return to the sites that better serve their needs. With the development of recommender systems, various kinds of recommendation techniques have been proposed, including collaborative filtering (CF) [1–3], content-based filtering [4, 5],  *K*-nearest neighbor (*K*-NN) [6–8], diffusion approach [9, 10], and spectral analysis [11, 12]. A collaborative filtering approach builds a model to predict what users will like according to their similarity to other users based on collecting and analyzing a large amount of information on users' behaviors, activities, or preferences. Content-based filtering methods are based on information about and characteristics of the products that are going to be recommended. They try to recommend products that are similar to those that a user liked in the past.  *K*-NN is one of the most fundamental and simple classification methods for classifying objects based on the properties of their closest neighbors in the feature space. In  *K*-NN, an object is classified through a majority vote of its neighbors, with the object being assigned to the class most common amongst its  *k*  nearest neighbors. Diffusion approach based on the mass diffusion principle generates the recommendation results for target users on a user-object bipartite network. Spectral analysis is a new recommendation algorithm that relies on the singular value decomposition (SVD) of the rating matrix.

Otherwise, more and more scholars apply clustering or classification techniques into recommendation methods in order to enhance the recommending accuracy. We know that users in a cluster will have similar interests; thus, if a product is selected by these users it will be suitable to the target user. It leads to more accurate recommendations. So, in this paper, we propose a novel collaborative filtering recommendation approach based on  *K*-means clustering algorithm. The reason why we choose  *K*-means is that it is the most popular class of clustering algorithms while simple and fast. However,  *K*-means algorithm highly depends on the initial states and always converges to the nearest local optimum from the starting position of the search. Therefore, in the process of clustering, we use artificial bee colony (ABC) algorithm to overcome these problems. And then we adopt the modified cosine similarity considering products' popularity degrees and users' preference degrees to compute the similarity between users in the same clusters. Finally, we generate the recommendation results for target users. Detailed numerical analysis on a benchmark dataset *MovieLens* and a real-world dataset indicates that our new collaborative filtering approach based on users clustering algorithm outperforms many other methods.

The main contribution of this paper is summarized as follows: we propose a novel collaborative filtering recommendation approach to generate good recommendation results for target users. We import  *K*-means clustering algorithm into it in order to enhance the accuracy of recommendations. And we modify the standard cosine similarity for the sake of more accuracy and diversity of the recommendation results. The remainder of this paper is organized as follows. In Section 2, a review of related work is given. In Section 3, our recommendation model and new collaborative filtering method based on users clustering using artificial bee colony algorithm are described.  Section 4 provides experimental results and analysis of the proposed method on a benchmark dataset and a real-world dataset. Finally, we draw conclusions in Section 5.

## 2. Related Work 

In this section, we will introduce some good collaborative recommendation techniques and then describe certain relevant conceptions.

Liu et al. [13] proposed a novel method to compute the similarity between congeneric nodes on bipartite network. They considered the influence of a node's degree and then presented a modified collaborative filtering (MCF) to substitute the standard cosine similarity. Kim et al. [14] proposed a collaborative approach to user modeling for enhancing personalized recommendations to users. Their approach first discovered some useful and meaningful user patterns and then enriched the personal model with collaboration from other similar users. López-Nores et al. [15] presented a new strategy called property-based collaborative filtering (PBCF) to address problems of recommender systems by introducing a new filtering strategy, centered on the properties that characterized the items and the users. Tsai and Hung [16] assessed the applicability of cluster ensembles to collaborative filtering recommendation. They used two well-known clustering techniques and three ensemble methods. The experimental results based on the *MovieLens* dataset showed that cluster ensembles could provide better recommendation performance than a single clustering technique in terms of recommendation accuracy and precision. Altingovde et al. [17] explored an individualistic strategy which initially clustered the users and then exploited the members within clusters, but not just the cluster representatives, during the recommendation generation stage. They provided an efficient implementation of this strategy by adapting a specifically tailored cluster-skipping inverted index structure. Wu et al. [18] presented a novel modified collaborative recommendation method called div-clustering to cluster Web entities in which the properties were specified formally in a recommendation framework, with the reusability of the user modeling component considered. Choi and Suh [19] proposed a new similarity function in order to select different neighbors for each different target item. In the new similarity function, the rating of a user on an item was weighted by the item similarity between the item and the target item. Gan and Jiang [20] proposed a network-based collaborative filtering approach to overcome the adverse influence of popular objects while achieving a reasonable balance between the accuracy and the diversity. Their method started with the construction of a user similarity network from historical data by using a nearest neighbor approach. Based on this network, they calculated discriminant scores for candidate objects and further sorted the objects in nonascending order to obtain the final ranking list. Bilge and Polat [21] proposed a novel privacy-preserving collaborative filtering scheme based on bisecting  *K*-means clustering in which they applied two preprocessing methods. The first preprocessing scheme dealt with scalability problem by constructing a binary decision tree through a bisecting  *K*-means clustering approach while the second produced clones of users by inserting pseudo-self-predictions into original user profiles to boost accuracy of scalability-enhanced structure.

### 2.1. *K*-Means Algorithm

*K*-means is a rather simple but well-known algorithm for grouping objects. Let *X* = {*x*_1_, *x*_2_,…, *x*_*n*_}  be a set of  *n*  objects. Each object can be thought of as being represented by some feature vector in a *p*-dimensional space. This algorithm starts by guessing  *k*  cluster centers and then iterates the following steps until convergence is achieved [22].

1. Clusters are built by assigning each element to the closest cluster center.
2. Each cluster center is replaced by the mean of the elements belonging to that cluster. The algorithm is described as follows.

Set dataset  *X* = {*x*_*i*_}, *i* = 1,2, 3,…, *n*, and assemble them as *k*  cluster, that is, to divide the dataset as the follows:

(1) $$\begin{array}{c} X=\cup_{j=1}^{k}C_{j},\;\;C_{j1}\cap C_{j2}=\emptyset ,\;j1\neq j2. \end{array}$$

*C*_*j*_  is the arbitrary cluster.

**Step 1**

Initialize cluster centers  *c*_*j*_, *j* = {1,…, *k*}.

**Step 2**

Assemble the dataset  *X*  by cluster centers, as

(2) $$\begin{array}{c} c_{j}:=\left\{x\left|||x-c_{j}||=\min\right|,x\in X\right\},\;j=1,2,\ldots ,k. \end{array}$$

In which  ||∗||  as some kind of norm, as clustering often be processed in Euclidean space in fact, the norm mentioned above often be set as 2-norm.  *c*_*j*_  is the center of the cluster  *C*_*j*_.

**Step 3**

Update cluster centers as follows:

(3) $$\begin{array}{c} c_{j}:=\left(\frac{1}{K_{j}}\right)\sum x\in C_{j},\;j=1,2,\ldots ,k, \end{array}$$

where  *K*_*j*_  is the number of data in cluster  *C*_*j*_.

**Step 4**

Stop. If the cluster centers do not change or the clustering converge towards some kind of value, the iteration is stop. For instance, the clustering converge condition meets a cost function  *f*_*i*_:

(4) $$\begin{array}{c} f_{i}=\frac{1}{k}\sum_{j=1\;}^{k}\sum_{x_{i}\in C_{j}}d\left(x_{i},c_{j}\right). \end{array}$$

Though the users similarity computation, we can find some products selected by the test users who have much similarity to the target user.

### 2.2. Artificial Bee Colony Algorithm

Artificial bee colony (ABC) is one of the most recently defined algorithms by Karaboga [23] in 2005, motivated by the intelligent behavior of honey bees. It is a very simple, robust, and population-based stochastic optimization algorithm. The performance of the ABC algorithm is compared with those of other well-known modern heuristic algorithms such as differential evolution (DE) and particle swarm optimization (PSO) on constrained and unconstrained problems [24–26]. In ABC algorithm, the colony of artificial bees contains three groups of bees: employed bees, onlookers, and scouts. A food source represents a possible solution to the problem to be optimized. The nectar amount of a food source corresponds to the quality of the solution represented by that food source. For every food source, there is only one employed bee. In other words, the number of employed bees is equal to the number of food sources around the hive. The employed bee whose food source has been abandoned by the bees becomes a scout.

The main steps of the algorithm can be described as follows.

**Step 1**

Generate the initial population of solutions by using a random approach. Let  *X*_*i*_^*j*^  represent the  *i*th feasible solution (food source). Each feasible solution is generated as follows:

(5) $$\begin{array}{c} X_{i}^{j}=X_{\min}^{j}\;+\text{rand}\left(0,1\right)\left(X_{\max}^{j}-{\;X}_{\min}^{j}\right), \end{array}$$

where  *X*_max⁡_^*j*^  and  *X*_min⁡_^*j*^  are the lower and upper bounds for the dimension  *j*, respectively.

**Step 2**

Produce new solutions for the employed bees, evaluate them, and apply the greedy selection process. The formula of the selection can be expressed as

(6) $$\begin{array}{c} V_{i}^{j}=X_{i}^{j}+\text{rand}\left[-1,1\right]\left(X_{i}^{j}-\;X_{k}^{j}\right), \end{array}$$

where  *j* ∈ {1,2,…, *D*}, *k* ∈ {1,2,…, SN}, and  *k* ≠ *i*. *D* is the dimension of the problem, representing the number of parameters to be optimized.  SN  is the number of the food sources and equals the number of employed bees or onlooker bees.

**Step 3**

Calculate the probabilities of the current sources with which they are preferred by the onlookers.

**Step 4**

Assign onlooker bees to employed bees according to probabilities, produce new solutions, and apply the greedy selection process. Formula (7) is used to calculate the probability value used by the onlooker bees for discovering promising regions in the search space:

(7) $$\begin{array}{c} p_{t}=\frac{\text{fi}{\text{t}}_{t}}{\sum_{j=1}^{\text{SN}}\text{fi}{\text{t}}_{j}}, \end{array}$$

where  fit_*t*_ = 1/(1 + *f*_*i*_)  and  *f*_*i*_  is the objective function.

**Step 5**

If the search times *Bas* of an employed bee is more than threshold *limit*, stop the exploitation process of the sources abandoned by bees and send the scouts in the search area for discovering new food sources, randomly.

**Step 6**

Memorize the best food source found so far.

**Step 7**

If the termination condition is not satisfied, go to Step 2; otherwise, stop the algorithm.

## 3. Collaborative Recommendation Approach Based on Users Clustering

**Figure 1:**
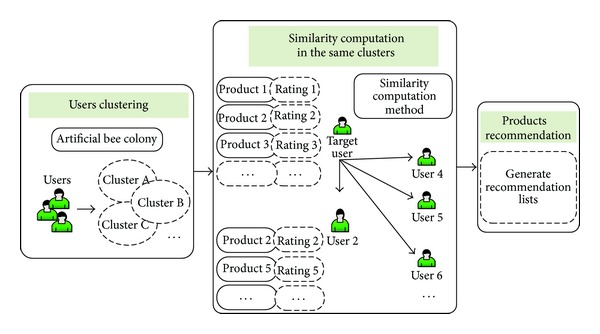
The framework of recommendation model based on users clustering.

In this section, we describe the proposed collaborative filtering recommendation approach based on  *K*-means clustering algorithm. In the process of clustering, we use ABC algorithm to overcome the local optimal problem of the  *K*-means clustering algorithm. After that we adopt the modified cosine similarity which considers products' popularity degrees and users' preference degrees to compute the similarity between users in the same clusters. Finally, we generate a recommendation list made up of these products (objects) and then recommend them to the target users.  Figure 1 shows the framework of our proposed recommendation model.

There are three phases in this framework.

*(1) User Clustering.* In order to enhance the accuracy of recommendation results, we use  *K*-means clustering method to cluster users before recommending. As we know that users in a cluster will have similar interests, thus, if a product is selected by these users, it will be suitable to the target user. It leads to more accurate recommendations. For the sake of overcoming the local optimal problem of this clustering method, we bring in ABC algorithm. The steps are shown in Section 3.1.

*(2) Similarity Computation.* Firstly, we give some definitions. We assume that there is a recommendation model which consists of  *m*  users and  *n*  objects, and each user has selected some objects. The relationship between users and objects can be described by a bipartite network. Let  *U* = {*u*_1_, *u*_2_,…, *u*_*m*_}  denote users set and  *O* = {*o*_1_, *o*_2_,…, *o*_*n*_}  denote objects set, and the recommendation model can be fully described by an  *m* × *n*  adjacency matrix  *A* = {*a*_*ij*_}, where  *a*_*ij*_ = 1  when object  *j*  is selected by user  *i*; otherwise,  *a*_*ij*_ = 0. After that we use modified collaborative filtering method to compute the similarity between users. The detailed process of this computation is described in Section 3.2. 

*(3) Products Recommendation.* In the previous phase, we compute the similarity between target user and others based on the influence of each object node including popular degree and preference degree. In this step, we calculate the value of comprehensive preference degree of each product unselected by the target user. Afterwards, the products with high comprehensive preference degree are used to compile a recommendation list in descending order. At last we recommend top  *L*  products to the target user. In general, the number  *L*  is no more than 100. 

### 3.1. *K*-Means Clustering Method Using ABC Algorithm

In the process of users clustering, we use ABC algorithm to overcome the local optimal problem of  *K*-means. As we know, in traditional  *K*-means algorithm, users need to preset the  *k*  value and centre points which will often have a big influence on cluster results. In our clustering method, we use ABC algorithm to determine the optimal value of centre points. According to many literatures in setting value of  *k*, we predict  *k*  following a principle which meets$\;k\leq (\sqrt{n}-1)/2\;\;($consider that the optimal value  *k*  satisfies the conditions of  *k*_opt_ ≤ *k*_max⁡_  and$\;k_{\max}\leq \sqrt{n})$. In this clustering algorithm, the solutions equate to the cluster centers. The steps of  *K*-means method using ABC algorithm are described as follows.

**Step 1**

Generate the initial population of solutions (the number of solutions is less than the predicted value  *k*  mentioned above) and the maximal search times *limit*. Let the  *K*-means' cost function (4) be as the objective function.

**Step 2**

Produce new solutions for the employed bees, evaluate them, and apply the greedy selection process.

**Step 3**

Calculate the probabilities of the current sources with which they are preferred by the onlookers.

**Step 4**

Assign onlooker bees to employed bees according to probabilities, produce new solutions, and apply the greedy selection process. That is making a clustering iteration of  *K*-means.

**Step 5**

If the search times *Bas* of an employed bee is more than threshold *limit*, stop the exploitation process of the sources abandoned by bees and send the scouts in the search area for discovering new food sources, randomly.

**Step 6**

Memorize the best food source found so far.

**Step 7**

If the termination condition is not satisfied, go to Step 2; otherwise, stop the algorithm.

**Step**

Determine the optimal centre points. Then assemble the dataset by these cluster centers and get the final results.

### 3.2. Similarity Computation of Modified Collaborative Filtering

After users clustering, we will use modified collaborative recommendation approach to generate recommendation results for target users. Traditional collaborative filtering method usually adopts the standard cosine similarity or Pearson correlation to compute the similarity between two users. For arbitrary users  *u*_*i*_  and  *u*_*j*_, the number of common objects shared by them can be expressed as

(8) $$\begin{array}{c} c_{ij}=\sum_{l=1}^{n}a_{li}a_{lj}. \end{array}$$

Generally, for standard cosine similarity computation, let  *s*_*ij*_  denote the similarity between  *u*_*i*_  and  *u*_*j*_  and *k*(*u*_*i*_)/*k*(*u*_*j*_)  denote the degree of the user  *u*_*i*_/*u*_*j*_  namely, how many objects are collected by this user. So we can formulate the expression as:

(9) $$\begin{array}{c} s_{ij}=\frac{c_{ij}}{\sqrt{k\left(u_{i}\right)k\left(u_{j}\right)}}=\frac{\sum_{l=1}^{n}a_{li}a_{lj}}{\sqrt{k\left(u_{i}\right)k\left(u_{j}\right)}}. \end{array}$$

The problem of (9) is that it has not taken into account the influence of an object's degree, so that objects with different degrees have the same contribution to the similarity. If users  *u*_*i*_  and  *u*_*j*_  both have selected object  *o*_*l*_; that is to say, they have a similar preference for the object  *o*_*l*_. In addition, in real recommender system, the similarity computation between two users is not simple but influenced by many factors. So we need to improve the traditional collaborative filtering method in order to fit the complex conditions. In fact, the similarity between two users should be somewhat relative to their degrees and preference degrees; that is, each object node's degree and preference degree are related to its popular degree and corresponding users' comments or ratings, respectively.

We assume that the similarity computation on user-object bipartite networks is affected by an influence degree  1/(1 + |*v*_*li*_ − *v*_*l*__*j*_|)*k*(*o*_*l*_), where  *v*_*li*_/*v*_*lj*_  represents the preference degree that object  *o*_*l*_  obtained from user  *u*_*i*_/*u*_*j*_,  *k*(*o*_*l*_)  denotes the degree of the object  *o*_*l*_; namely, how many users select this object. Accordingly, the contribution of object  *o*_*l*_  to the similarity  *s*_*ij*_  should be negatively correlated to its degree  *k*(*o*_*l*_)  and the difference of its preference degrees evaluated by different users. The formulation of  *s*_*ij*_  can be expressed as

(10) $$\begin{array}{c} s_{ij}=\frac{1}{\sqrt{k\left(u_{i}\right)k\left(u_{j}\right)}}\sum_{l=1}^{n}\frac{a_{li}a_{lj}}{\left(1+\left|v_{li}-{v_{l}}_{j}\right|\right)k\left(o_{l}\right)}. \end{array}$$

Though the users similarity computation, we can find the products unselected by target user but selected by test users who have much similarity to target user. Let  *p*_*ij*_  represent the preference degree of object  *o*_*j*_  obtained from target user  *u*_*i*_. The formulation of  *p*_*ij*_  can be expressed as

(11) $$\begin{array}{c} p_{ij}=\sum_{l=1,l\neq i}^{m}s_{il}a_{jl}. \end{array}$$

In the process of recommendation, we get the elements of  *p*_*ij*_  uncollected by target user and then sort them in descending order, as target user prefers the objects in the top, so we recommend top  *L*  objects to this user.

### 3.3. Recommendation Performance Metrics

In this paper, we adopt some widely used metrics to measure the accuracy and diversity of the presented recommendation method, in which accuracy is the most important aspect in evaluating the recommendation algorithmic performance. They are five metrics: Ranking score, precision, recall, intrasimilarity, Hamming distance. The first three are used to test accuracy and the rest are used to test diversity. The detailed descriptions of these metrics are as follows. 

*(1) Ranking Score.* For an arbitrary user  *u*_*i*_, if the recommended object  *o*_*j*_  (*o*_*j*_  is an uncollected object for  *u*_*i*_)  is ranked in  *R*_*ij*_  position in the ordered recommendation list  *L*_*i*_, we can formulate the expression as  *r*_*ij*_ = *R*_*ij*_/*L*_*i*_. For example, if the length of  *L*_*i*_  is 200, namely, there are 200 uncollected objects for  *u*_*i*_  and  *o*_*j*_  is in the 10th place, we can get the value of  *r*_*ij*_ = 10/200 = 0.05.  The average of  *r*_*ij*_  over all user-object pairs in the test set is denoted by  〈*r*〉, which can be used to evaluate the algorithmic accuracy. The smaller the ranking score is, the higher the algorithmic accuracy will be. 

*(2) Precision.* It is defined as the ratio of the number of recommended objects collected by users appearing in the test set to the total number of recommended objects. This measure is used to evaluate the validity of a given recommendation list. The precision can be formulated as  *a*/*L*, in which  *a*  represents the number of recommended products collected by users appearing in test set, and  *L*  is the total number of recommended products. In general, the number of recommended products is no more than 100. 

*(3) Recall.* It is defined as the ratio of the number of recommended objects collected by users appearing in the test set to the total number of the objects actually collected by these users. The larger recall corresponds to the better performance. The Recall can be formulated as  *a*/*M*, in which  *a*  represents the number of recommended products collected by users appearing in test set, and  *M*  is the total number of these users' actual buying. 

*(4) Intrasimilarity.* It evaluates the similarity between objects inside users' recommendation lists. A good recommendation algorithm is expected to give fruitful recommendation results and has the ability to guide or help the users exploit their potential interest fields. Therefore, it calls for a lower intrasimilarity. There are many similarity metrics between objects. Here we adopt the widely used one, that is, cosine similarity to measure objects' similarity. For two objects  *o*_*i*_  and  *o*_*j*_, their similarity is defined as

(12) $$\begin{array}{c} S_{ij}=\frac{\sum_{l=1}^{m}a_{li}a_{lj}}{\sqrt{k\left(o_{i}\right)k\left(o_{j}\right)}}. \end{array}$$

For an arbitrary user  *u*_*l*_, the number of recommendation objects is  *L*. Firstly, we need to calculate  *L*(*L* − 1)/2  couple of objects' similarity and then average these values to get  *I*_*l*_ = 〈*S*_*ij*_〉. Finally, we use the mean value of  *I*  of the overall users to measure the diversity in recommendation lists. 

*(5) Hamming Distance.* It can measure the strength of personalization. If the overlapped number of objects in  *u*_*i*_  and  *u*_*i*_'s recommendation lists is  *Q*, their Hamming distance is

(13) $$\begin{array}{c} H_{ij}=1-\frac{Q}{L}. \end{array}$$

Generally speaking, a more personalized recommendation list should have long Hamming distances to other lists. Accordingly, we use the mean value of Hamming distance *S* = 〈*H*_*ij*_〉  of the overall user-user pairs to measure the strength of personalization. 

## 4. Experimental Results and Analysis

In this section, we design two groups of experiments for testing the performance of our method.  (1)  The first group: we verify the validity of the proposed  *K*-means method using ABC algorithm.  (2)  The second group: we measure the performance of our recommendation approach based on the improved  *K*-means. All algorithms mentioned below are implemented in Matlab 7.9 using computer with Intel Core 2 Duo CPU E7500, 2.93 GHz, 4 GB Memory. The operating system of the computer is Windows XP.

### 4.1. Validity of *K*-Means Clustering Method Using ABC

In fact, Karaboga and Ozturk [27] have demonstrated that ABC algorithm could efficiently be used for multivariate data clustering such as  *K*-means. In their work, 13 classification problems from the UCI database, which was a well-known database repository, were used to evaluate the performance of the ABC algorithm. The results showed that  *K*-means method using ABC algorithm outperforms it using PSO algorithm in 12 problems, whereas PSO algorithm's result was better than that of ABC algorithm only for one problem (the glass problem) in terms of classification error. Moreover, the average classification error percentages for all problems were 13.13% for ABC and 15.99% for PSO.

Given this, we compare the proposed method with traditional  *K*-means in an additional metric  *D*/*L*, where  *D*  is the internal distance in clusters and  *L*  is the distance between clusters. The smaller the value of  *D*/*L*  is, the better the clustering quality will be.  Table 1 shows the results of the comparison.

### 4.2. Performance of Recommendation Approach Based on the Improved *K*-Means

In testing recommendation algorithmic performance, we use a benchmark dataset *MovieLens *[28] and a real-world dataset. The *MovieLens *dataset consists of 1682 movies, 943 users, and 100,000 ratings. Each user has rated at least 20 movies by using a discrete number on the scale of 1 to 5. In this dataset, there are three kinds of information tables: demographic information about the users, information about the items (movies), and scores about the movies. The real-world data is extracted from a well-known Chinese online bookstore. It contains 86,500 users, providing 1,360,780 ratings about 250,400 books. Each user has an account which records some information from him. The information mainly contains region, age, income, and so on. The ratings are expressed on a discrete number on the scale of 1 to 10. Before experimenting, we need to preprocess these two datasets. For *MovieLens*, only the links with ratings no less than 3 are considered and  *v*_*li*_ = {3,4, 5}. For the real-world dataset, only the links with ratings no less than 5 are considered and  *v*_*li*_ = {5,6, 7,8, 9,10}. Besides, we divide the each processed dataset into two parts: the training set which contains 90% of the data and the remaining 10% of the data for the test.

Firstly, the clustering method requires the number of clusters to be provided as an input. So, we set  *k* = 15  for the *MovieLens* and  *k* = 146  for the real-world dataset (on the basis of$\;k=(\sqrt{n}-1)/2$). Hereinafter, we name our method as cluster-based CF. As known to all, in the steps of generating recommendations, we need to set a certain length of recommendation list  *L*  considering recall and precision measures. Generally, merchants recommend no less than 50  (*L*)  products to target users through recommender systems.

We compare cluster-based CF with three other widely used recommendation algorithms: CF, MCF, and NNCosNgbr [29] in all five metrics. We summarize the algorithmic performance in Table 2 for the *MovieLens* and Table 3 for the real-world dataset.

Comparing cluster-based CF with the standard CF, as is seen in Table 2 in the condition of recommendation number  *L* = 50, the ranking score can be further reduced by 22.4%, the precision can be further increased by 11.8% and with MCF, the ranking score can be reduced by 18.2%, and the precision can be further increased by 8.3%. Similarly, the proposed algorithm has lower ranking score and higher precision than NN-CosNgbr algorithm. For the rest of metrics, our algorithm is also the best. Although the real-world dataset is similar to* MovieLens*, it is much sparse. So we set the number of recommended products no less than 50.

Table 3 shows that the proposed algorithm exceeds other three algorithms in all the five criterions: lower ranking score, higher precision, bigger recall, lower intrasimilarity, and larger hamming distance.

At last, for an online recommender system, we need to consider the processing time and memory consumption of its recommendation algorithm. In contrast, the process of users clustering increases computational complexity of cluster-based CF. But in the process of similarity calculation, the computing range is significantly reduced that leads to lower computational complexity. Therefore, the computational complexity of cluster-based CF is almost close to traditional CF's. Likewise, the memory store of cluster-based CF is similar to traditional CF's. In addition, the online recommending platform is therefore needed to have efficient access to, at least, two types of resources: data and computing processors. For small scale data recommending tasks, a single desktop computer, which contains hard disk and CPU processors, is sufficient to fulfill the recommending goals. For medium scale data recommending tasks, data are typically large (and possibly distributed) and cannot be fit into the main memory. Furthermore, if a new object is added to the collection or a new user is registered to the recommender system, our algorithm can properly generate recommendation results for it through the clustering computing before recommending.

## 5. Conclusions

Recommendation model helps users to find out their potential future likes and interests. It recommends good products to users and satisfies the users' demands as far as possible. The application of clustering techniques reduces the sparsity and improves the scalability of the recommendation model since the similarity can be calculated only for users in the same clusters. An excellent recommendation method meets high accuracy and certain diversity and can enhance the quality of personalized service.

In this paper, we propose a novel collaborative filtering recommendation approach based on  *K*-means clustering algorithm. Firstly, we use artificial bee colony (ABC) algorithm to overcome  *K*-means algorithm's problems. And then we adopt the modified cosine similarity considering products' popularity degrees and users' preference degrees to compute the similarity between users in the same clusters. Finally, we generate the recommendation results for target users. Detailed numerical analysis on a benchmark dataset *MovieLens* and a real-world dataset indicates that our new collaborative filtering approach based on users clustering algorithm outperforms many other recommendation methods.

Concerning future work, we will research in the following aspects: how to improve the  *K*-means clustering algorithm or adopt other superior clustering algorithms to increase the validity and precision of cluster results. Introduce techniques of implicit information extraction of users and design reasonable clustering partition strategy which considers more actual influence factors; How to keep the robustness of recommendation algorithm when it meets hostile attacks. Hostile attacks mean that someone makes hostile and large number of invalid ratings or evaluations to recommender systems. Through hostile attacks, it is possible to affect the availability of the recommender systems.

## Figures and Tables

**Table 1:** The ratio of *K*-means using ABC to traditional *K*-means in metric *D*/*L*.

	Number of instances	Number of attributes	*k*	D/L
				Ratio of *K*-means using ABC to traditional *K*-means
Balance	625	4	3	0.873
Cancer	569	30	2	0.862
Cancer-Int	699	9	2	0.871
Credit	690	15	2	0.891
Dermatology	366	34	6	0.886
Diabetes	768	8	2	0.879
Ecoli	327	7	5	0.881
Glass	214	9	6	0.895
Heart	303	75	2	0.868
Horse	364	27	3	0.859
Iris	150	4	3	0.860
Thyroid	215	5	3	0.867
Wine	178	13	3	0.876

What we can know from this table is that our method is better than traditional *K*-means and it has more applicable cluster results.

**Table 2:** Algorithmic performance for *MovieLens *dataset. The ranking score, precision, recall, intrasimilarity, Hamming distance are corresponding to *L* = 30, 40, and 50. Each number presented in this table is obtained by averaging over five runs, each of which has an independently random division of training set and test.

Algorithms	Ranking score	Precision	Recall	Intrasimilarity	Hamming distance
*L* = 30					
CF	0.148	0.077	0.321	0.330	0.704
MCF	0.131	0.087	0.360	0.306	0.751
NN- CosNgbr	0.124	0.085	0.352	0.311	0.744
cluster-based CF	0.109	0.098	0.393	0.279	0.796

*L* = 40					
CF	0.137	0.071	0.332	0.338	0.698
MCF	0.121	0.080	0.373	0.317	0.743
NN- CosNgbr	0.120	0.079	0.369	0.320	0.736
cluster-based CF	0.105	0.089	0.405	0.288	0.790

*L* = 50					
CF	0.125	0.066	0.343	0.347	0.692
MCF	0.110	0.072	0.385	0.330	0.735
NN- CosNgbr	0.109	0.070	0.381	0.334	0.729
cluster-based CF	0.097	0.078	0.417	0.301	0.776

**Table 3:** Algorithmic performance for the real-world dataset. The ranking score, precision, recall, intrasimilarity, Hamming distance are corresponding to *L* = 50, 60, and 70. Each number presented in this table is obtained by averaging over five runs, each of which has an independently random division of training set and test.

Algorithms	Ranking score	Precision	Recall	Intrasimilarity	Hamming distance
*L* = 50					
CF	0.052	0.035	0.171	0.364	0.479
MCF	0.043	0.042	0.182	0.328	0.527
NN- CosNgbr	0.042	0.041	0.184	0.332	0.523
cluster-based CF	0.034	0.047	0.201	0.272	0.614

*L* = 60					
CF	0.046	0.032	0.185	0.385	0.461
MCF	0.040	0.039	0.194	0.342	0.506
NN- CosNgbr	0.039	0.038	0.196	0.348	0.504
cluster-based CF	0.031	0.043	0.209	0.281	0.603

*L* = 70					
CF	0.041	0.030	0.202	0.403	0.457
MCF	0.035	0.036	0.216	0.362	0.502
NN- CosNgbr	0.034	0.035	0.217	0.364	0.499
cluster-based CF	0.027	0.040	0.231	0.293	0.592
